# Microfluidics-Based Time-Resolved Fluorescence Immunoassay for the On-Site Detection of Aflatoxins B1 Zearalenone and Deoxynivalenol in Cereals

**DOI:** 10.3390/foods11091319

**Published:** 2022-04-30

**Authors:** Xu Wang, Disha Lu, Qingfeng Huang, Jinyi Yang

**Affiliations:** Guangdong Provincial Key Laboratory of Food Quality and Safety, College of Food Science, South China Agricultural University, Guangzhou 510642, China; 18574851733@163.com (X.W.); louteksa@163.com (D.L.); hqfeng23@163.com (Q.H.)

**Keywords:** aflatoxins B1, zearalenone, deoxynivalenol, monoclonal antibody, microfluidics, time-resolved fluorescence immunoassay, cereals

## Abstract

The primary pollutants in cereal products are aflatoxins B1 (AFB1), zearalenone (ZEN), and deoxynivalenol (DON). In this study, anti-AFB1 MAb (4C9), anti-ZEN MAb (2A3), and anti-DON MAb (1F10) were developed and used in time-resolved fluorescence immunoassay microfluidics to determine AFB1, ZEN, and DON in agricultural products. The linear range for AFB1, ZEN, and DON were 0.05~2.2 μg/kg, 1.45~375.75 μg/kg, and 11.1~124.2 μg/kg, respectively. In maize, the recoveries of AFB1/ZEN/DON were 92~101%, 102~105%, and 103~108%, respectively. High-performance liquid chromatography and the proposed approach had a good correlation. Time-resolved fluorescence immunoassay microfluidics is a highly efficient and sensitive field detection method for fungal toxins in agricultural products.

## 1. Introduction

Mycotoxins are a group of toxic secondary metabolites produced by fungi in high-temperature, high-humidity environments [1]. To date, more than 30 fungal strains have been identified as producing toxins that are poisonous to both people and animals. Among them, Aflatoxins B1 (AFB1), Zearalenone (ZEN), and Deoxynivalenol (DON) have been the main contaminants in cereal products [2,3]. The mycotoxins’ high-temperature tolerance resulted in residue on the agricultural products. These properties have serious implications for human health [4,5,6]. A single fungus can contaminate a wide range of agricultural products and foods, and food products are commonly contaminated by a mycotoxin symbiosis due to a diversified diet [7]. Mycotoxins not only cause a lot of food waste and serious food poisoning, but also damage the structural function of cells, and cause inhibition of the synthesis of various enzymes, nucleic acids, and proteins in the organism, which can seriously affect the survival and health of humans and animals when they ingest these contaminated foods [8,9,10]. Mycotoxin contamination has therefore become an important, and a difficult, issue in countries around the world, and has sparked continued concern worldwide. Strict national standards have been set to limit the levels of mycotoxins in cereals, to ensure food safety and human health. As a result, several countries have set limits on the levels of AFB1, ZEN, and DON in food and animal feed. The MRLs of AFB1, ZEN, and DON, based on the European Pharmacopoeia, are 2, 100, and 750 μg/kg, respectively. According to the U.S. FDA, the maximum residue limits for AFB1 and DON are 20 and 1000 μg/kg, respectively, and ZEN does not meet the requirements. According to the National Standard of the People’s Republic of China, the maximum residue limits of AFB1, ZEN, and DON are 0.5, 60, and 1000 μg/kg respectively [11]. There is, therefore, an urgent need to establish multiplexed methods for the determination of mycotoxins.

At present, the methods of multiple detection of mycotoxins include immunoassay and instrumental analysis. Instrumental methods include high-performance liquid chromatography (HPLC), gas chromatography-mass spectrometry (GC-MS/MS), and liquid chromatography-tandem mass spectrometry (LC-MS/MS) [12,13,14]. Field testing for mycotoxins is accurate and reliable, but it requires expensive equipment, complex sample pre-treatment methods, and specialized personnel, which severely limits its use. The enzyme-linked immunosorbent assay (ELISA) and the colloidal gold immunoassay (GICA) are the most extensively utilized immunoassay procedures. Immunoassay methods have the advantages of simplicity of operation, rapid results, and low cost, making them ideal methodologies for large-scale screening and monitoring in the field. However, most ELISAs used for mycotoxin analysis are single analyte formats [15,16,17]. Most colloidal gold assays for multiplex analysis of mycotoxins are only qualitative or semi-quantitative [18,19,20]. In conclusion, for these reported multiplex immunoassays to be used as a practical method for field monitoring, there are two constraints that must be solved: (a) single detection capability; and (b) low sensitivity.

To boost the sensitivity of the assay, we created the AFB1/ZEN/DON monoclonal antibody in this work. To enhance the multiplex detection capability, this study designed a self-driven, microfluidics-based immunosensor that can simultaneously detect three analytes. The proposed method was using time-resolved fluorescent microspheres as labels, which is more advantageous in a larger specific surface area, Stokes shift, and longer fluorescence lifetime. This method has the potential to be a tool for the detection of a wide range of fungal toxins.

## 2. Experimental Section

### 2.1. Reagents and Instruments

Aflatoxin B1 (AFB1), aflatoxin B2 (AFB2), aflatoxin G1 (AFG1), aflatoxin G2 (AFG2), aflatoxin M1 (AFM1), aflatoxin M2 (AFM2), ZEN, α-zearalenol (α-ZOL), α-zearalanol (α-ZER), β-zearalenol (β-ZOL),β-zearalanol (β-ZER), deoxynivalenol (DON), 3-acetyldeoxynivalenol (3Ac-DON), 15-acetyldeoxynivalenol (15Ac-DON), nivalenol, NIV, fumonisin B1 (FB1), T-2 toxin and ochratoxin A (OTA), polyethylene glycol (PEG), complete and incomplete Freund’s adjuvants, bovine serum albumin (BSA) and ovalbumin (OVA), 1-(3-dimethylaminopropyl)-3-ethyl-carbo-diimide hydrochloride (EDC), and N-hydroxysuccinimide (NHS) by Sigma–Aldrich (St. Louis, MO, USA). Fluorescent microspheres were purchased from Suzhou Vdo Biotech Co., Ltd., (Suzhou, China). PMMA solepate and PET cover plate were supplied by HiComp Microtech Co., Ltd., Inc., (Suzhou, China). Carbodiimide (CDI), butaneboronic acid (BBA), 4-dimethylamio-pyridine (DMAP), carboxymethoxylamine hemihydrochloride (CMO), *N*,*N*-dimethylforma-mide (DMF), tween-20, pyridine, methanol (MeOH), and ethyl acetate were obtained from Damao Chemical Reagent Co., Ltd., (Tianjin, China). RPMI-1640 cell culture medium, HAT, and HT media supplement (50×) were purchased from Sigma (Shanghai, China). Bal b/c female mice were supplied by the Guangdong Medical Experimental Animal Centre and raised at South China Agriculture University Animal Centre. All other reagents were of analytical reagent grade or higher purity.

Quant Quantification Fluorescence Reader (Hi Comp Microtech Co., Ltd., Shenzhen China). Absorbances of ELISA were measured on a Multiskan micro-plate reader (Thermo Lab systems, Philadelphia, PA, USA). Absorbances of antibody concentrations were measured on a NanoDrop 2000c Ultraviolet spectrometer (Thermo Fisher Scientific Co., Ltd., Waltham, MA, USA).

The LC-MS assay was conducted using an Agilent 6400 series LC system and an ECLIPS PLUS C18 (2.1 × 100 mm, 1.8 µm) (Agilent Technologies, CA, USA) with a Triple Quadrupole Mass Spectrometer.

### 2.2. Preparation of Antigens

**Figure 1:**
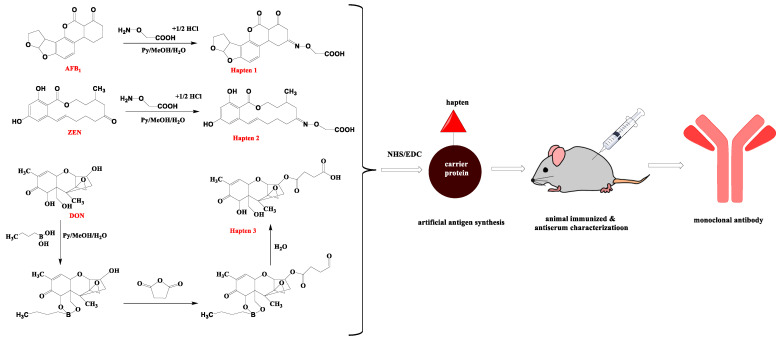
The schematic of developed MAb for AFB1, ZEN, and DON. Hapten 1, Hapten 2, and Hapten3 are hapten of AFB1, ZEN, and DON, respectively. The semi-antigens were coupled with BSA to obtain immunogens, and antibodies were obtained by immunization in mice.

Hapten 1 (Figure 1): AFB_1_-CMO. The Aflatoxin B_1_ (10 mg, 0.03 mmol), Carboxymeth-oxylamine hemi hydrochloride) (20 mg, 0.15 mmol), methanol (6.7 mL), pyridine (1.65 mL), and ultrapure water (1.65 mL) were put into a round-bottom flask, and stirred at 50 °C for 8 h. The residue in the bottle was dissolved with pure water and trichloromethane, and then dried under a stream of nitrogen. The product was recrystallized into a white solid. Using the active ester technique, Hapten 1 was conjugated to BSA for immunogen, and to OVA for antigen coating. Amounts of 0.025 mmol hapten, 0.0375 mmol EDC, and 0.0375 mmol NHS were mixed in 0.6 mL DMF, and agitated for 4 h. Afterwards, 50 mg protein (dissolved in 5 mL CB) was added to the mixture, and the pH was adjusted to 9.6 using 3 M NaOH. The solution was agitated overnight. The solution was then dialyzed for 3 days with 0.01 M PBS, before being kept at 20 °C until use.

Hapten 2: other than using AFB1 instead of ZEN, Hapten 2 was synthesized using the same method as Hapten 1.

Hapten 3: the DON (10 mg, 0.03 mmol), pyridine (12 mL), and BBA (40 mg, 0.39 mmol) were packed into a round-bottom flask, and stirred at 25 °C for 12 h. Then, 75 mg of succinic anhydride and 29 mg of DMAP were stirred at 60 °C for 2 h. The reaction was then stopped by adding 2.5 mL of ultrapure water, and dried under nitrogen. The residue in the bottle was dissolved with pure water and trichloromethane, and then dried under a stream of nitrogen. Then the pure product was recrystallized into white solid.

Using the active ester technique, Hapten 3 was conjugated to BSA for immunogen.

Hapten 3 was coupled with OVA (DON-OVA) for coating antigens. DON (2 mg) and CDI (12 mg) were mixed in anhydrous dimethylformamide (0.5 mL), and the mixture was agitated for 1.5 h at 37 °C. The reaction solution was then dropped into CBS (3 mL) containing OVA (10 mg), and reacted at 37 °C for 20 h. The solution was then dialyzed for 3 days with 0.01 M PBS (5 L), before being kept at 20 °C until use.

### 2.3. Production of MAb

For the first time, female BALB/c mice (6–8 weeks old) were used for immunization. 80 g of antigen was emulsified and mixed with complete Freund’s adjuvant (1:1, *v/v*), and multiple subcutaneous injections were performed. This was followed by immunization every 21 days using incomplete Freund’s adjuvant emulsified with 30 g of immunogen (1:1 *v/v*), for a total of four immunizations. Blood was drawn from a tail vein after the fifth inoculation, and the antibody titer and inhibitory rates were measured using an ELISA test.

The mouse spleen cell and the SP2/0 murine myeloma cells were washed three times in RPMI 1640 medium. The SP2/0 cells and spleen cells were then combined (5:1), and hybridomas were created using 1 mL PEG at 37 °C. The RPMI 1640 medium was then added to prevent fusing. The hybridoma cells were grown in five 96-well plates using HAT medium for the first five days, then switched to HT media for the last five days. On the tenth day, ic-ELISA was used to screen the medium of each well. Limit dilution was performed on the well with the highest titer and inhibition. Limiting dilution was used to subclone the designated cell group five times (0.6 cell per well). The hybridoma developed was used to prepare ascites. Protein G resin was used to extract and purify the monoclonal antibody (MAb) from ascites. The MAb was collected and kept at −20 °C.

### 2.4. Indirect Competitive ELISA

The antigen was lysed using coating buffer (0.05 M CBS, pH 9.6), and dropped into 96-well plates (100 μL/well), then incubated for 2 h at 37 °C. After three washes with washes buffer (0.01 M PBS containing 0.05% Tween-20), 200 mL of blocking buffer (0.05 M CBS containing 2% BSA) was added to each well and incubated for 2 h at 37 °C overnight. Following three washes, 50 μL Mab (gradient dilution) and 50 μL DON standard solution were added to each well, and incubated for 30 min at 37 °C. Then, each well was filled with 100 μL of horseradish peroxidase-labeled Goat anti-mouse IgG antibody (diluted 1: 3000 in PBS) and incubated for 30 min at 37 °C. Following four washes, each well was filled with 100 L of HRP substrate, and incubated for 15 min at 37 °C in the dark. After that, 50 μL of stop solution (2 M sulfuric acid) was administered to each well. Finally, the optical density (OD) of each well at 450 nm was determined using a microplate reader.

### 2.5. Characterization of MAb

Using an isotype analysis kit, antibody isotypes were determined based on the binding of a mouse monoclonal antibody to a single isotype-specific antibody. Serial dilutions were made, of the encapsulated antigen and antibody; the horizontal coordinate was the logarithm of the antibody concentration (mM), the vertical coordinate was the matched absorbance, and the curve was plotted. Based on the curve’s 50% inhibitory concentration (IC50), cross-reactivity (CR) was calculated and used to determine the specificity of the monoclonal antibody.

### 2.6. Antibody Specificity Determination

The cross-reactivity (CR) of the anti-AFB1/ZEN/DON MAb was utilized as a measure of its specificity. To calculate the IC_50_ (50% inhibitory concentration) of each major mycotoxin contaminant (aflatoxin B1, aflatoxin G1, aflatoxin B2, aflatoxin G2, aflatoxin M1, aflatoxin M2, α-ZOL, β-ZOL, β-ZAL, α-ZAL, 3-AcDON, NIV, 15-AcDON, ochratoxin A, T-2 toxin, and fumonisin B1), inhibition curves were fitted using ELISA data. The following equation was used to compute the CR:

(1) $$\mathrm{CR}=\frac{{\mathrm{IC}}_{50}\;\mathrm{of}\;{\mathrm{AFB}}_{1}/\mathrm{ZEN}/\mathrm{DON}}{{\mathrm{IC}}_{50}\;\mathrm{of}\;\mathrm{common}\;\mathrm{mycotoxin}\;\mathrm{contaminant}}\;\times \;100\%$$

### 2.7. Prepare TRFMs-Probes

To begin, 100 μL of fluorescent microspheres and 400 µL of activation buffer (0.05 M BB, pH 8.0) were vortexed for 1 min to completely mix them. 5 μL NHS and 30 μL EDC (10 mg/mL) were reacted in TRFMs solution at 25 °C for 15 min to activate the carboxyl groups on the surface. After centrifuging the solution at 10,000× *g* for 10 min at 10 °C, the supernatant was discarded. Then, 50 μL of 0.5 mg/mL monoclonal antibody was added to the reaction buffer (0.05 M HEPES, pH 6.0) for 2 h. After centrifugation of the solution at 10,000× *g* for 10 min at 10 °C, the supernatant was discarded.

The precipitates were then sonicated for 1 min at 100 W using 0.5 mL of closure buffer (0.05 M BB, pH 8.0, containing 5% BSA, 1% casein, 0.1% polyethylene glycol 2000, 0.05% Tween-20), and reacted for 1 h. The precipitate was then sonicated using 0.25 mL of preservation buffer (0.05 M Tris, pH 8.0, containing 1% BSA, 0.2% glycine, 13% alginate, 2% sucrose, 0.05% Tween-20) for 1 min at 100 W and stored at 4 °C until use.

### 2.8. Assembly of the Microfluidic Chip

The microfluidic chip was composed of two components: a cover plate made of polyethylene terephthalate, and a soleplate coated with AFB1-OVA, ZEN-OVA, DON-OVA, and chicken IgY. Three different coating antigens were sprayed onto the detection channel to form the three-test area (T-1, 2, 3). The microfluidic chip was dried at 37 °C for 60 min, and stored in a desiccator. After cleaning with PBS buffer and pure water, 2 times each, respectively, 2 min/time, the chip was put in the drying oven at 37 °C for 20 min until the surface of chip was dry. The chip film was applied flat to the chip substrate, and completely enclosed.

### 2.9. Sample Pretreatment

Samples were collected at three points on the grain sample bag and mixed uniformly. Deionized water containing 75% alcohol was chosen as the extraction solvent. 5 g of the powder was put into a tube with 25 mL of extraction solvent. The centrifuge tubes were shaken for 10 min, and then centrifuged at 8000× *g* for 15 min; ic-ELISA and microfluidics were used to identify the results.

### 2.10. Microfluidics Assay Procedure

**Figure 2:**
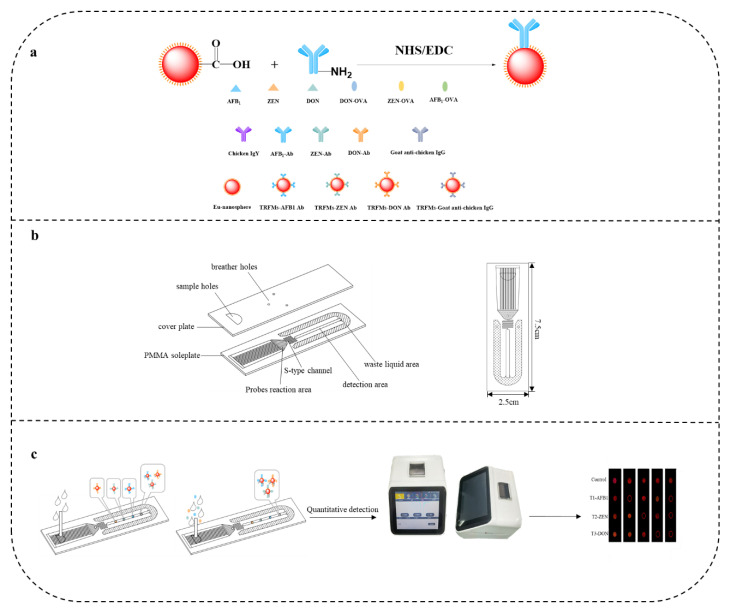
Schematic diagrams of the microfluidic chip for AFB_1_/ZEN/DON, and interpretation of test results: (**a**) principles of immunoprobe preparation; (**b**) schematic diagram of microfluidic chip; (**c**) detection principle of microfluidics chip; the EM wavelength of fluorescent microspheres was 365.0 nm, the EM wavelength was 650 nm, and the scanning range was from 500.0 nm to 700.0 nm.

**Figure 3:**
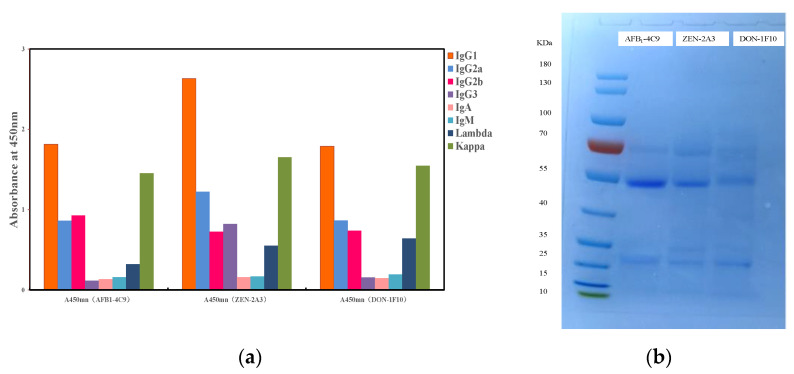
(**a**) Isotype determination of MAb; (**b**) The electropherogram of Mab.

A capillarity microfluidic chip was designed and manufactured to match with the fluorescence quantitative detector, as shown in Figure 2 and Figure 3; the microfluidic was 7.5 cm long and 2.5 cm wide, and consisted of two parts. The PMMA base plate had four functional units: a probe reaction area, an S-shaped channel, a detection area, and a waste area. The cover plate was a viscous polyethylene terephthalate (PET) membrane, consisting of sample holes and ventilation holes. The function of the probe reaction area in the lower cover was to allow the sample to react more fully with the probe. The S-shaped channel was to control the liquid flow rate to allow the sample to flow evenly into the detection area. The detection area was embedded with biological materials that could react with the probe to generate fluorescent signals. The waste liquid area was to collect the waste liquid after the reaction. The sample hole on the top cover was used for drip sample addition. The ventilation holes were to keep the liquid flowing normally. As the sample probe mix was fully reacted in the reaction zone, and flowed through the S-shaped channel to the detection zone, the free monoclonal antibody TRFMs probe competed with the target analyte to bind to the encapsulated antigen in the detection zone, and the goat anti-chicken IgG TRFMs probe binded to the encapsulated antigen in the control zone; it reacted with the encapsulated antigen in the detection zone, generating a fluorescent signal, and finally flowed into the waste zone. The sample was detected qualitatively, based on the fluorescence signal using the eye under UV light, and quantitatively by detecting the fluorescence intensity information using a quantitative fluorescence reader.

## 3. Results and Discussion

### 3.1. The Mab’s Characterization

After mouse cell fusion and limiting dilution, three monoclonal antibodies were isolated. These three monoclonal antibodies were antibody 4C9 for AFB_1_, antibody 2A3 for ZEN, and antibody 1F10 for DON. The isotype analysis kit revealed that the MAb generated belonged to the IgG subclass (Figure 3a). The electropherogram revealed distinct bands of heavy chain (about 55 kDa) and light chain (approximately 25 kDa) MAb (Figure 3b). The AFB1/ZEN/DON MAb concentration was 8.91/10.57/13.55 mg/mL, as determined by a NanoDrop ultraviolet spectrophotometer.

### 3.2. Antibody Specificity Determination

ELISA was used to detect the Mab’s cross-reactivity with other common mycotoxins. As shown in Table 1, mycotoxins containing similar structures displayed cross-reactivities. There was no cross-reaction with the other chemicals tested.

### 3.3. Indirect Competitive ELISA

**Figure 4:**
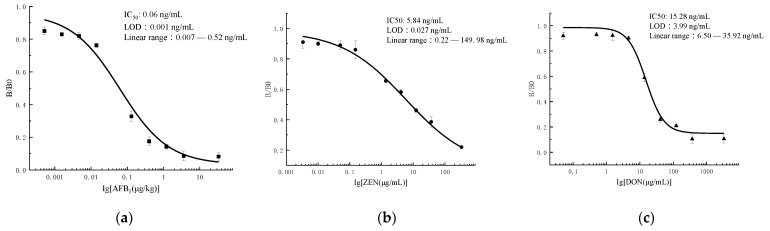
The inhibition curve of ic-ELISA: (**a**) AFB1; (**b**) ZEN; (**c**) DON (AFB1-OVA, ZEN-OVA, and DON-OVA were diluted and added to the wells of microtiter plates, and the coating buffer was 0.05 M carbonate buffer (pH 9.6). The reaction was carried out using 50 μL of MAb and HRP- IgG (1:5000) at 30 min, 37 °C).

The ic-ELISA parameters are listed in Table 2 and Figure 4a–c. The procedures are as follows: the connection between optical density (OD) and drug concentration was plotted to construct standard curves, and LOD and IC50 were determined for the three antibodies; the standard curve for AFB1 monoclonal antibodies was y = 0.03363 + (0.95753 − 0.03363)/(1 + (x/0.062)^0.65049^), the IC_50_ was 0.06 ng/mL, the LOD was 0.001 ng/mL, and the linear range was 0.0074~0.527 ng/mL; the standard curve for ZEN monoclonal antibodies was y = 0.08325 + (0.98334 − 0.08325)/(1 + (x/5.849)^0.42732^), the IC_50_ was 5.84 ng/mL, the LOD was 0.027 ng/mL, and the linear range was 0.22~149.98 ng/mL; the standard curve for DON monoclonal antibodies was y = 0.14943 + (0.98566 − 0.14943)/(1 + (x/15.28)^1.62245^), the IC_50_ was 15.28 ng/mL, the LOD was 3.99 ng/mL, and the linear range was 6.50~35.92 ng/mL.

### 3.4. Optimization of the Microfluidics

#### 3.4.1. Optimization of the Microspheres

**Figure 5:**
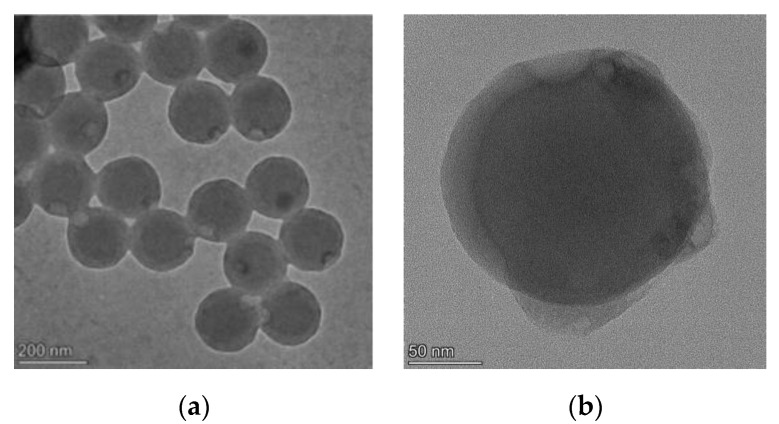
Scanning electron microscope image of fluorescent microsphere. (**a**) Scanning electron microscope image of 200 nm size; (**b**) Scanning electron microscope image of 50 nm size.

The diameter of time-resolved fluorescent microspheres (TRFMs) has different characteristics, according to previous reports. Microspheres with a particle size of 200 nm are lighter in color, and have a shorter detection range compared to 100 nm microspheres. Microspheres with a particle size of 300 nm have the highest fluorescence intensity compared to 200 nm microspheres, but the detection time is longer, and fluorescent microspheres with a sensitivity less than 200 nm cannot meet the requirements of rapid detection systems. Therefore, we labeled the MAbs with red microspheres with a particle size of 200 nm (Figure 5).

#### 3.4.2. Coating Buffer Selection

**Figure 6:**
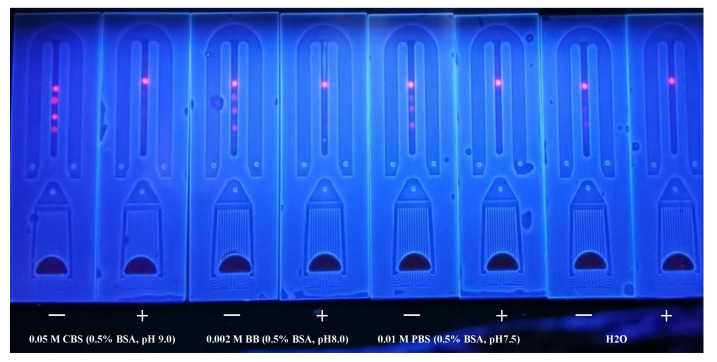
Different buffers for the coating buffer.

To dilute MAb, three antigen dilution buffers were used: 0.05 M CBS (0.5% BSA, pH 9.0); 0.002 M BB (0.5% BSA, pH 8.0); 0.01 M PBS (0.5% BSA, pH 7.5); and H_2_O. The results (Figure 6) showed that 0.05 M CBS reached the necessary fluorescence intensity and inhibitory effect for each line. As a result, we settled on 0.05 M CBS as the buffer for further investigation.

#### 3.4.3. Coating Antigen

**Figure 7:**
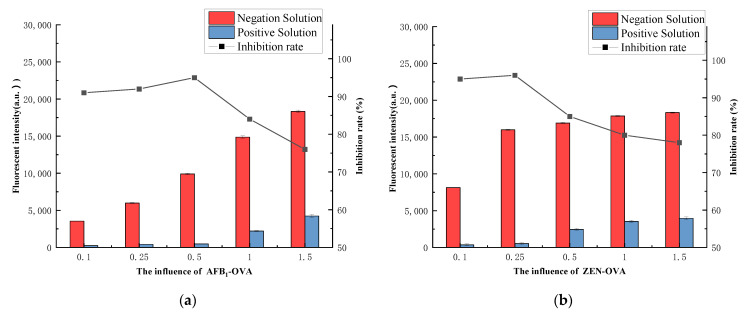

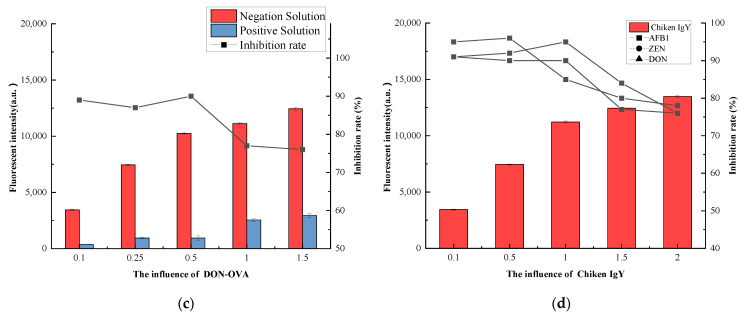
The coating antigens amount: (**a**) AFB_1_; (**b**) ZEN; (**c**) DON; (**d**) Chicken IgY.

Coated antigens have a direct impact on detection performance. When the coating antigen is too low, it reduces the fluorescence intensity of the detection area, resulting in reduced sensitivity. Finally, as shown in Figure 7a–c, the addition ratios of Chicken IgY, AFB1-OVA, ZEN-OVA, and DON-OVA were adjusted and Chicken IgY (1 mg), AFB1-OVA (0.5 mg), ZEN-OVA (0.25 mg) and DON-OVA (0.5 mg) were selected as the most suitable addition amounts for antigen encapsulation.

### 3.5. Sensitivity of the Microfluidics

**Figure 8:**
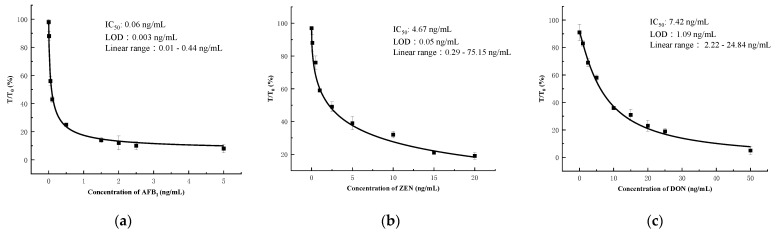
The calibration curve of mMicrofluidics: (**a**) AFB_1_; (**b**) ZEN; (**c**) DON.

**Figure 9:**
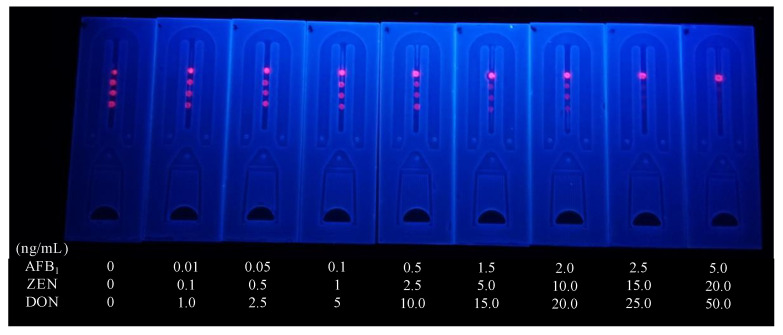
The Sensitivity analysis of Microfluidics: AFB_1_; ZEN; DON.

Using 0.01 M PBS, different concentrations of AFB1, ZEN, and DON were produced and utilized to assess the detection performance of microfluidics. The visible limit of detection (vLOD) for the eye was defined as the sample concentration at which the fluorescence disappeared from the test area. A fluorescence reader was used to measure color intensity in quantitative assays. The calculated lower limit of detection (cLOD) was defined as the concentration at 10% inhibition (IC10) of the standard curve. The linear range was defined as the concentration at the standard curve IC20 to IC80. T/T0 was defined as the ratio of the fluorescence intensity of the test area of the standard sample to the fluorescence intensity of the blank sample. After determining the T/T0 value of the unknown sample using a fluorescence reader, the concentrations of AFB1, ZEN, and DON were calculated. The results for AFB1 are shown in Figure 8a: the AFB_1_ calibration curve was y = 6.30618 + (99.0788–6.30618)/(1 + (x/0.06979)^0.74859^); the vLOD was 1.5 ng/mL; the cut-off value was 2.5 ng/mL; the cLOD was 0.003 ng/mL; and the theoretical linear range was 0.01~0.44 ng/mL. The results for Zen are shown in Figure 8b: the Zencalibration curve was y = −19.84726 + (97.08969 − 19.84726)/(1 + (x/4.67164)^0.49902^); the vLOD was 5.0 ng/mL; the cut-off value was 15.00 ng/mL; the cLOD was 0.05 ng/mL; and the theoretical linear range was 0.29~75.15 ng/mL. The results for DON are shown in Figure 8c: the DON calibration curve was y = −1.56823 + (91.41032 − 1.56823)/(1 + (x/7.42751)^1.14818^); the vLOD was 10 ng/mL; the cut-off value was 25 ng/mL; the cLOD was 1.09 ng/mL; and the theoretical linear range was 2.22~24.84 ng/mL. The actual test is shown in Figure 9.

### 3.6. Spiked Sample Analysis

Samples were collected by Winsor Food Group Co., Ltd.; these samples included both positive and negative samples. As shown in Table 3, after sample pretreatment, the AFB1, ZEN, and DON sample linear range was 0.05~2.2 μg/kg, 1.45~375.75 μg/kg, and 11.1~124.2 μg/kg (samples exceeding the linear range were diluted and tested). The test was repeated three times for each sample, and the AFB1/ZEN/DON concentrations in the samples were calculated using the calibration curve. In maize, the recoveries of AFB1/ZEN/DON were 92~101%, 102~105%, and 103~108%, respectively, compared to the recoveries of ic-ELISA (100~107%, 101~103%, 100~105%), which indicated no significant difference between the two methods.

### 3.7. Natural Sample Analysis

Eight different natural samples of corn, wheat, and brown rice were analyzed using the microfluidics and an LC-MS method. As shown in Table 4, the data obtained by the two methods were consistent.

### 3.8. Comparison of the Microfluidics

Table 5 summarizes multiplex detection for mycotoxins in the last five years. It can be concluded that most of the current detection targets are 2–3 mycotoxins, and the detection methods are mainly microfluidic or LFIA. Desktop readers or handheld readers were used for quantitative testing. The time-resolved fluorescence immunoassay based on microfluidic technology has advantages in terms of multi-detection capability, portability, and rapid quantification of assay results compared to multiplex assays reported in the last five years.

## 4. Conclusions

In this paper, three MAbs against AFB1, ZEN, and DON were generated and characterized. Based on the MAbs, a capillarity of microfluidics rapid determination, sensitivity detection, and reliability of AFB_1_, ZEN, and DON residues in agricultural products was developed. The limits of detection were 0.003/0.05/1.09 ng/mL, respectively, of AFB1, ZEN, and DON in 10 min by Quant Quantification Fluorescence Reader; and the visual limits of detection were 1.5/5.0/10 ng/mL, respectively, of AFB1, ZEN, and DON by eyes. Compared with LFIA, microfluidics can detect three residues simultaneously. Microfluidic assembly is simple, using easy to obtain materials, no NC membrane, glass cellulose and other materials; it is a new method of immunochromatographic assay. The method provides qualitative and quantitative results for the rapid detection of AFB1, ZEN, and DON in samples. Overall, the multiplex, high-sensitivity detection strategy developed in this study demonstrates the promise of rapid, portable field monitoring and analysis of naturally occurring mycotoxins in cereals.

## Figures and Tables

**Table 1:** IC50 and cross-reactivity (CR) of anti-AFB1/ZEN/DON MAb against related mycotoxins. (*n* = 3).

Mycotoxin Analyte	Structure	IC50 (ng/mL)	CR (%)
AFB1	-	0.08	100%
	AFB2	0.67	12%
	AFM1	1.11	7%
	AFM2	10.7	1%
	AFG1	11.56	1%
	AFG2	15.53	1%
	ZEN, DON, FB1, OTA, T-2	>10,000	<0.1
ZEN	-	6.41	100%
	ZAN	28.82	22%
	α-ZOL	56.24	11%
	β-ZOL	53.31	12%
	α-ZAL	44.28	14%
	β-ZAL	48.22	13%
	AFB1, DON, FB1, OTA, T-2	>10,000	<0.1
DON	-	16.31	100%
	3-AcDON	66.28	25%
	15-AcDON	>10000	<0.1
	NIV	>10,000	<0.1
	ZEN, AFB1, FB1, OTA, T-2	>10,000	<0.1

**Table 2:** Indirect competitive ELISA for AFB1/ZEN/DON monoclonal antibodies.

Target Analyte	AFB1	ZEN	DON
Coating antigen	AFB1-OVA	ZEN-OVA	DON-OVA
Coating concentration (μg/mL)	0.42	0.34	0.46
Coating buffer	0.05 M carbonate buffer (pH 9.6)		
Coating condition	14 h, 4 °C		
MAb	Anti-AFB1 MAb	Anti-ZEN MAb	Anti-DON MAb
Standard analyte	AFB1	ZEN	DON
Competition condition	30 min, 37 °C		
HRP- IgG dilution	1:5000		
HRP-IgG incubation condition	30 min, 37 °C		

**Table 3:** Comparison of microfluidic and ic-ELISA spiked recovery (*n* = 3).

Analyte	Spiked Level (µg/kg)	Microfluidics Detection Level (µg/kg)			Visual	Ic-ELISA Detection Level (µg/kg)		
		Mean ± SD	Recovery Rate (%)	CV (%)		Mean ± SD	Recovery Rate (%)	CV (%)
AFB1	0	ND	NC	NC	− − −	ND	NC	NC
	5	4.75 ± 0.01	95	1	± ± ±	5.33 ± 0.35	107	7
	25	22.9 ± 1.18	92	5	+ + +	25.08 ± 0.21	100	1
	50	50.65 ± 1.13	101	2	+ + +	51.1 ± 0.39	102	1
ZEN	0	ND	NC	NC	− − −	ND	NC	NC
	50	52.28 ± 2.92	105	6	± ± ±	50.66 ± 1.20	101	2
	250	258.05 ± 8.19	103	3	+ + +	257.83 ± 11.11	103	4
	500	510.00 ± 6.68	102	1	+ + +	513.32 ± 8.17	103	2
DON	0	ND	NC	NC	− − −	ND	NC	NC
	50	54.20 ± 2.31	108	4	± ± ±	55.35 ± 2.56	111	5
	250	258.55 ± 3.45	103	1	+ + +	261.65 ± 5.34	105	2
	500	512.68 ± 4.99	103	1	+ + +	501.65 ± 5.13	100	1

ND, not detectable; NC, not calculated; −, negative: the concentration of AFB_1_/ZEN/DON was below 0.5/1.5/20 μg/kg; ±, weakly positive: the concentration of DON was in the range of 2.5~12.5/7.5~75/50~125 μg/kg; +, positive: the concentration of AFB_1_/ZEN/DON exceeded 12.5/75/125 μg/kg.

**Table 4:** Naturally sample analysis with Microfluidics (*n* = 6).

Sample	LC-MS (μg/kg)			Microfluidics (μg/kg)					
	AFB1	ZEN	DON	AFB1	AFB1-Visual	ZEN	ZEN-Visual	DON	DON-Visual
1 * corn	1.31	388.33	715.54	1.48	± ^a^	- ^c^	+	-	+
2 * corn	2.44	133.11	189.15	256	±	-	+	-	+
3 * corn	1.53	298.54	85.07	1.66	±	-	+	-	+
4 * wheat	52.81	634.36	2258.37	-	+ ^b^	-	+	-	+
5 * wheat	3.62	1105.28	3125.8	4.02	±	-	+	-	+
6 * wheat	85.31	18.36	276.81	-	+	18.66	±	-	+
7 * brown rice	2.11	33.84	71.26	2.46	±	-	+	70.55	±
8 * brown rice	2.32	18.42	65.54	2.64	±	19.16	+	66.39	±

^a^, weak positive. ^b^, positive. ^c^, Result cannot be calculated.

**Table 5:** Comparison of the Microfluidics.

Method	Target Analytes	Test Sample	Mechanical System	vLOD	cLOD	Quantitative Device	Reference
Microfluidics	T2/OTA/AFB1/ ZEN	Corn, wheat	Syringe pump	-	0.12/0.03/1.24/0.58 μg/kg (sample)	Fluorescent strip reader	[21]
Microfluidics	AFB1/OTA/ DON	Maize, wheat	Magnetic valves	-	3/100/100 μg/kg (sample)		[22]
Microfluidics	AFB1/OTA/ DON	feed	Syringe valves	-	<40/0.1~0.2/<10 ng/mL (buffer)	Smart phone	[23]
QDs-LFIA	FB1/AFB1/ OTA	Maize, rice, wheat	capillarity	-	1.58/0.002/0.059 ng/mL (buffer)	ESE-Quant LFR Fluorescence reader	[24]
TRFMs-LFIA	ZEN/AFB1	Maize	capillarity	-	0.07/0.05 ng/mL (buffer)	Homemade portable Fluorescence	[25]
GNPs-LFIA	FB1/ZEN/ DON	Maize, wheat	capillarity	60/6/12.5 ng/mL (buffer)	-	-	[26]
Multicolor-LFIA	ZEN/T-2/AFB1	Maize, feed	capillarity	2/30/0.5 ng/mL (buffer)	-	-	[27]
SPR chip	T-2/HT-2, DON	Wheat, maize	-	-	12.1/29/31 ng/mL (sample)	SPR reader-	[28]
Microchip coupled with ELISA and electrochemical	ZEA	Corn	-	-	<1 μg/L	MK3 microplate reader-	[29]
Microfluidics	AFB1/ZEN/ DON	Corn, wheat, rice	capillarity	1.5/5.0/10 ng/mL (buffer)	0.003/0.05/1.09 ng/mL (buffer)	Fluorescent strip reader	present work

-, unavailable.

## Data Availability

The datasets generated for this study are available on request to the corresponding author.
